# HIV infection and survival in Hodgkin lymphoma: a real-world analysis of a multicenter research network

**DOI:** 10.1093/jncics/pkag076

**Published:** 2026-08-13

**Authors:** Shih-Ping Lin, Tsung-Han Yen, Jian-Ri Li, Cheng-Wei Chou

**Affiliations:** Department of internal medicine, Taichung Veterans General Hospital, Taichung, Taiwan; Department of Post-Baccalaureate Medicine, College of Medicine, National Chung Hsing University, Taichung, Taiwan; Division of Urology, Department of Surgery, Taichung Veterans General Hospital, Taichung, Taiwan; Division of Urology, Department of Surgery, Taichung Veterans General Hospital, Taichung, Taiwan; Department of Post-Baccalaureate Medicine, College of Medicine, National Chung Hsing University, Taichung, Taiwan; Division of Medical Oncology, Department of Oncology, Taichung Veterans General Hospital, Taichung, Taiwan

## Abstract

**Background:**

Despite effective antiretroviral therapy, people living with human immunodeficiency virus (HIV) (PLWH) remain at a higher risk of developing Hodgkin lymphoma (HL). Further, survival disparities persist despite similar treatment recommendations.

**Methods:**

Herein, we present a global retrospective cohort study including data of adults (≥18 years) diagnosed with HL between 2015 and 2025 from the TriNetX database to evaluate the survival outcomes of Hodgkin lymphoma in HIV-infected vs non-HIV-infected patients and analyze the associated risk factors. Patients were stratified according to HIV status and matched 1:1 for age, sex, race, and comorbidities. Overall survival (OS) was analyzed using Kaplan-Meier and Cox proportional hazards models.

**Results:**

A total of 927 matched HIV-infected and non-HIV-infected patients were included. Overall, HIV-positive status was associated with significantly inferior OS (HR 1.42; 95% CI 1.10-1.84; P = .033). Among PLWH, cerebrovascular disease, hepatitis C virus infection, and chronic kidney disease independently predicted increased mortality. In a matched subgroup of patients treated with doxorubicin, bleomycin, vinblastine, and dacarbazine (ABVD) chemotherapy (n = 154 per group), OS did not differ significantly between the HIV-positive and HIV-negative groups (HR 1.37; 95% CI 0.73-2.56; P = .247).

**Conclusion:**

In conclusion, HIV-infected patients with HL experienced worse OS compared with non-HIV-infected individuals, but the outcomes were comparable when treated with ABVD chemotherapy, highlighting the importance of optimal treatment delivery and comorbidity management in PLWH.

## Introduction

Since the introduction of active antiretroviral therapy (ART), the HIV infection has become a chronic and manageable condition. Beginning in 2015, both the US Department of Health and Human Services (DHHS) and the European acquired immunodeficiency syndrome Clinical Society (EACS) have recommended that all people living with HIV (PLWH) should receive ART.^1^^,^^2^ The survival rate of PLWH receiving ART has improved dramatically over the past 2 decades. The life expectancy of PLWH who began ART early and maintain viral suppression is now comparable to that of the general population.^3^ However, even in the era of ART, PLWH still face a 1.6 to 1.7-fold higher risk of developing cancer than the general population.^4^^,^^5^ Factors influencing the risk of cancer in PLWH include immunosuppression and chronic inflammation caused by HIV infection. In addition, PLWH are more susceptible to cancer-related risk behaviors and coinfections with other oncogenic viruses.^6^

The incidence and morbidity of AIDS-defining cancers (ADCs) have decreased due to ART, whereas the morbidity and mortality associated with non-AIDS-defining cancers (NADCs) have increased.^7^ In the United States, cancer-related mortality among PLWH was 386.9/100 000 person-years, with 9.2% and 5.0% of deaths attributed to NADCs and ADCs, respectively. Among NADCs, Hodgkin lymphoma is one of the leading causes of cancer-related death.^8^ Furthermore, 3.79% of patients with Hodgkin lymphoma test HIV-positive at the time of diagnosis.^9^ Currently, PLWH with Hodgkin lymphoma are treated similarly to patients without HIV, and the prognosis of Hodgkin lymphoma in PLWH approaches that of the general population.

As per the French ANRS-CO16 Lymphovir cohort, the prognosis for PLWH in the modern ART era was comparable to that of non-HIV-infected patients.^10^ A study conducted in the United Kingdom reported a complete chemotherapy response rate of 74% in HIV-infected patients compared to 79% in non-HIV-infected patients.^11^ However, according to the National Cancer Database in the United States, among patients who received chemotherapy, the HIV-positive status was not significantly associated with increased mortality. The poorer survival outcomes observed in HIV-associated Hodgkin lymphoma are largely attributed to lower rates of chemotherapy administration.^12^ Despite the availability of ART, PLWH continue to experience worse cancer outcomes, partly due to inequalities in cancer treatment. Compared to individuals with Hodgkin lymphoma without HIV, PLWH are 1.39 times more likely to not receive cancer treatment.^13^

Real-world data on the survival outcomes among PLWH with Hodgkin lymphoma during the ART era remain limited.^14^ This study aimed to evaluate the survival outcomes of patients with Hodgkin lymphoma among HIV-infected vs non-infected patients and analyze the associated risk factors using a global health research database.

## Methods

### Database

We conducted a global retrospective cohort study by collecting data from all patients with Hodgkin lymphoma using the multicenter research network TriNetX (Cambridge, Massachusetts, United States). TriNetX is a global federated platform that enables the analysis of anonymized clinical data provided by academic institutions, hospitals, and healthcare systems worldwide. This platform offers comprehensive patient-level data, including demographics, diagnostic codes, treatments, prescriptions, and laboratory parameters. The data used in this study was collected on July 10, 2025 from the TriNetX network. This study was approved by the Institutional Review Board of Taichung Veterans General Hospital (IRB No.: CF35731C). The requirement for informed consent was waived because only de-identified data were used.

### Patient selection, definitions, and study outcomes

Patients aged ≥18 years with Hodgkin lymphoma were identified in the TriNetX database between January 1, 2015 and January 1, 2025. Hodgkin lymphoma was diagnosed based on the International Classification of Diseases, Tenth Revision (ICD-10) with the diagnostic code C81. Patients with a history of other malignancies were excluded based on the ICD-10 codes C00-C14, C15-C26, C30-C39, C43-C44, C45-C49, C50-C50, C51-C58, C60-C63, C64-C68, and C69-C72.

The patient population was divided into 2 cohorts based on HIV diagnosis. HIV infection was identified using the ICD-10 code B20. Patients diagnosed with HIV either before or within 6 months of their Hodgkin lymphoma diagnosis were included in the HIV-infected group, whereas those without an HIV diagnosis were assigned to the non-HIV group. Patients who received at least one cycle of doxorubicin, bleomycin, vinblastine, and dacarbazine (ABVD) were classified into the chemotherapy group. The study period began in 2015 as treatment guidelines recommended ART for all PLWH since that year. The index date was defined as the date of Hodgkin lymphoma diagnosis.

Demographics and underlying comorbidities were also examined. Demographic information included age at the index event (years), sex, and race. Underlying comorbidities were identified using ICD-10-CM diagnosis codes and included hypertension (ICD-10: I10-I1A), ischemic heart disease (ICD-10: I20-I25), cardiovascular disease (ICD-10: I60-I69), chronic lower respiratory disease (ICD-10: J40-J4A), diabetes mellitus (ICD-10: E08-E13), chronic hepatitis B virus infection (ICD-10: B18.1), chronic hepatitis C virus (HCV) infection (ICD-10: B18.2), and chronic kidney disease (CKD) (ICD-10: N18). First, we performed 1:1 propensity score matching based on age, sex, race, and comorbid conditions to compare the overall survival (OS) rates between individuals who tested positive and negative for HIV infection, as well as among patients treated with ABVD in both the groups. The objective of this study was to compare the OS rates between the HIV-positive and HIV-negative groups with Hodgkin lymphoma. Furthermore, we analyzed the OS rates of patients with Hodgkin lymphoma who received chemotherapy in both the groups. The independent risk factors for mortality among HIV-infected patients with Hodgkin lymphoma were also evaluated.

### Statistical analyses

Baseline characteristics between the HIV-positive and HIV-negative groups were compared using the Student’s *t*-test for continuous variables and the chi-square test for categorical variables. OS was estimated using the Kaplan-Meier method, and survival distributions were compared using the log-rank test. Hazard ratios (HRs) and 95% confidence intervals (CIs) were calculated using Cox proportional hazard models. To address potential confounders that could bias the results, the cohorts were balanced using 1:1 greedy nearest-neighbor propensity score matching based on age, sex, race, and comorbid conditions. Subsequently, cox proportional hazards model analysis was used to identify independent risk factors for mortality among HIV-infected patients with Hodgkin lymphoma. Independent *t*-tests were used for continuous data, and chi-square tests were used for categorical data. Statistical significance was set at *P* < .05. All statistical analyses were performed using the TriNetX Analytics platform.

## Results

### Patient enrollment

**Figure 1.**
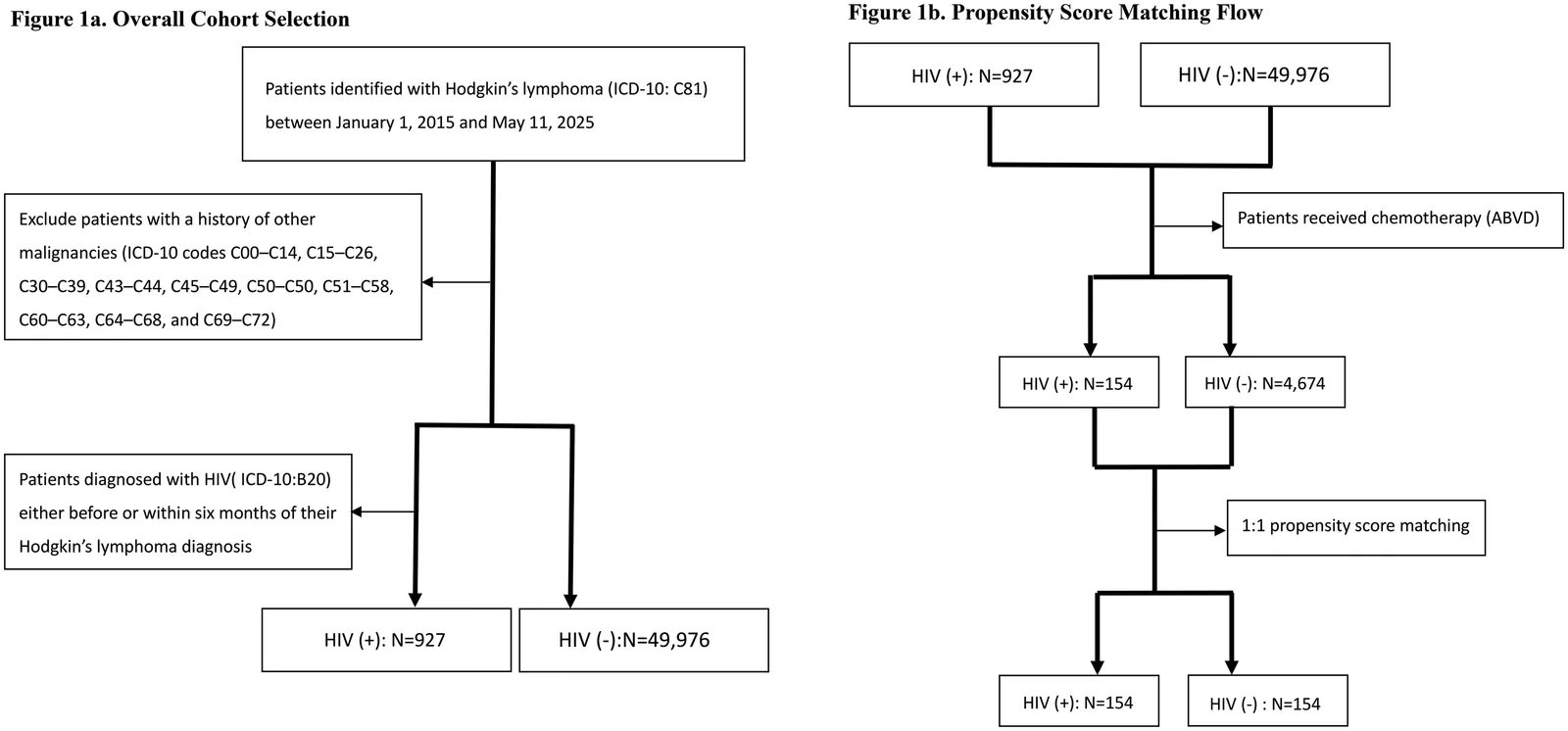
Study cohort selection and propensity score matching. Patients diagnosed with Hodgkin lymphoma (ICD-10 code: C81) between January 1, 2015 and January 1, 2025 were identified in the TriNetX database (*N* = 54 881). A total of 927 HIV-infected and 49 976 non-HIV-infected patients were eligible for inclusion (**A**). After 1:1 propensity score matching, 927 HIV-infected and 927 non-HIV-infected patients were included in survival analyses. Among the patients who received ABVD chemotherapy, 154 HIV-infected and 4674 non-HIV-infected patients were identified (**B**). After additional 1:1 propensity score matching, 154 HIV-infected and 154 non-HIV-infected patients were included in the chemotherapy subgroup analysis. ABVD = doxorubicin, bleomycin, vinblastine, and dacarbazine.

The flowchart of study enrolment is provided (Figure 1A and B). A total of 54 881 patients diagnosed with Hodgkin lymphoma between January 1, 2015 and January 1, 2025 were initially identified. After excluding patients with a history of other malignancies, 927 HIV-infected and 49 976 non-HIV-infected patients with Hodgkin lymphoma were identified (Figure 1A).

First, we performed 1:1 propensity score matching based on age, sex, and comorbidities, resulting in 927 matched patients in each group. We then analyzed the OS of patients with Hodgkin lymphoma in the matched HIV-positive and HIV-negative groups. The risk factors for mortality in the HIV-positive group (*N* = 927) were also evaluated.

Second, 154 HIV-infected and 4674 non-HIV-infected patients who received ABVD chemotherapy were included in the chemotherapy group (Figure 1B). Another 1:1 propensity score matching was performed, yielding a final cohort of 154 patients in each group. We then analyzed the OS of patients with Hodgkin lymphoma in the matched patients with and without HIV infection who received ABVD chemotherapy.

### Baseline characteristics of study participants

Demographic and clinical characteristics, including comorbidities, of the HIV-positive and HIV-negative groups before and after propensity score matching are summarized (Table 1). Prior to matching, patients with HIV infection were younger at diagnosis (mean age: 44.9 vs 46.9 years; *P *= .002) and more frequently male (80.5% vs 51.4%; *P *< .001) than non-HIV-infected patients. Racial distribution also differed, with most non-HIV-infected being White (56.0%), and the largest proportion of patients with HIV infection being Black or African American (43.0%). The HIV-positive group had higher prevalences of hypertension (32.1% vs 17.0%; *P *< .001), ischemic heart disease (9.4% vs 7.2%; *P *= .011), cardiovascular disease (6.1% vs 3.6%; *P *< .001), chronic lower respiratory disease (20.2% vs 9.6%; *P *< .001), diabetes mellitus (12.2% vs 7.7%; *P *< .001), hepatitis B virus infection (3.2% vs 0.3%; *P *< .001), hepatitis C virus infection (4.3% vs 0.5%; *P *< .001), and chronic kidney disease (10.5% vs 3.7%; *P *< .001). After propensity score matching, the baseline characteristics were well balanced between the groups. Approximately 43% of patients in both the groups were Black or African American, and the mean age was 46.9 ± 12.5 years in the HIV-positive group and 46.8 ± 12.7 years in the HIV-negative group, respectively. Patients from outside the United States accounted for only 9% of the cohort.

**Table 1.** Demographic and clinical characteristics and comorbidities of the HIV-positive and HIV-negative groups before and after matching.

	Before matching			After matching		
Demographics	HIV (+)	HIV (−)	*P*-value	HIV (+)	HIV (−)	*P-*value
*N*	927	49 976		927	927	
Age	46.9 ± 12.5	44.9 ± 19.5	.002	46.9 ± 12.5	46.8 ± 12.7	.852
Race						
White	340 (36.7%)	27 980 (56.0%)	<.001	340 (36.7%)	336 (36.2%)	.847
Black or African American	399 (43.0%)	4661 (9.3%)	<.001	399 (43.0%)	403 (43.5%)	.851
Others	57 (6.2%)	4882 (9.8%)	<.001	57 (6.2%)	57 (6.2%)	1.000
Unknown race	131 (14.1%)	12 453 (24.9%)	<.001	131 (14.1%)	131 (14.1%)	1.000
Sex						
Female	177 (19.1%)	23 105 (46.2%)	<.001	177 (19.1%)	180 (19.4%)	.860
Male	746 (80.5%)	25 686 (51.4%)	<.001	746 (80.5%)	743 (80.2%)	.861
Unknown sex	4 (0.4%)	1185 (2.4%)	<.001	4 (0.4%)	4 (0.4%)	1.000
Ethnicity						
Hispanic or Latino	151 (16.3%)	3206 (6.4%)	<.001	151 (16.3%)	152 (16.4%)	.950
Not Hispanic or Latino	565 (60.9%)	25 861 (51.7%)	<.001	565 (60.9%)	559 (60.3%)	.775
Unknown ethnicity	211 (22.8%)	20 909 (41.8%)	<.001	211 (22.8%)	216 (23.3%)	.783
Comorbid conditions						
Hypertension	298 (32.1%)	8475 (17.0%)	<.001	298 (32.1%)	299 (32.3%)	.960
Ischemic heart diseases	87 (9.4%)	3595 (7.2%)	.011	87 (9.4%)	80 (8.6%)	.570
Cerebrovascular diseases	57 (6.1%)	1800 (3.6%)	<.001	57 (6.1%)	55 (5.9%)	.845
Chronic lower respiratory diseases	187 (20.2%)	4810 (9.6%)	<.001	187 (20.2%)	187 (20.2%)	1.000
Diabetes mellitus	113 (12.2%)	3861 (7.7%)	<.001	113 (12.2%)	113 (12.2%)	1.000
Chronic hepatitis B infection	30 (3.2%)	128 (0.3%)	<.001	30 (3.2%)	29 (3.1%)	.895
Chronic hepatitis C infection	40 (4.3%)	238 (0.5%)	<.001	40 (4.3%)	37 (4.0%)	.727
Chronic kidney disease	97 (10.5%)	1849 (3.7%)	<.001	97 (10.5%)	93 (10.0%)	.759

### Impact of HIV status on overall survival in patients with Hodgkin lymphoma

**Figure 2.**
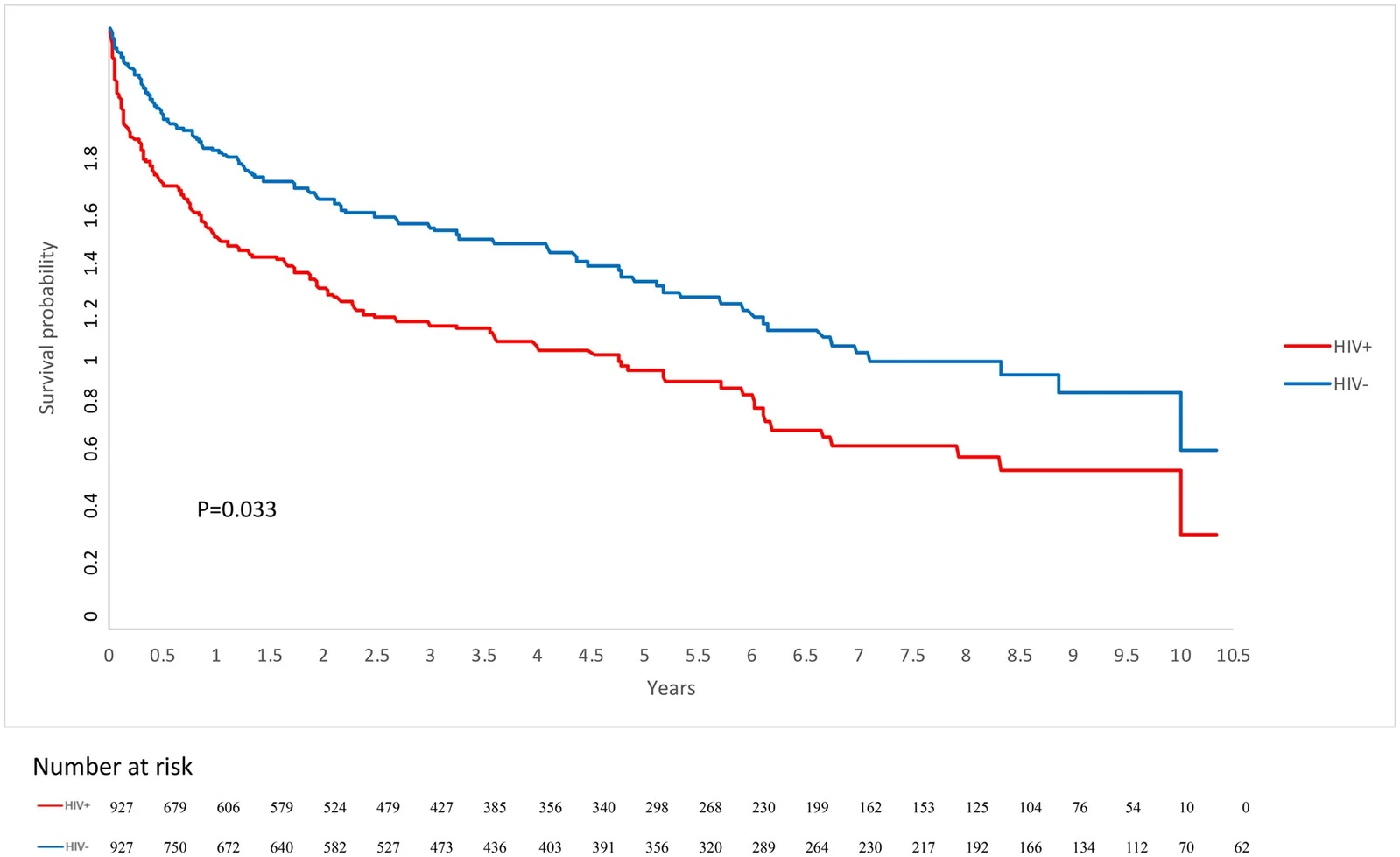
Kaplan-Meier analysis of overall survival of HIV-infected and non-HIV-infected patients with Hodgkin lymphoma after propensity score matching. A total of 927 HIV-infected and 927 non-HIV-infected patients with Hodgkin lymphoma were included after 1:1 propensity score matching.

After propensity score matching, 927 patients were analyzed in each group of patients with and without HIV. In terms of HIV-specific management, 72.5% (672/927) of the patients in the HIV-positive group had documented ART utilization or initiation within 6 months of their Hodgkin lymphoma diagnosis, indicating a high rate of ART. During the follow-up period, 138 and 102 deaths occurred in the HIV-positive and HIV-negative groups, respectively. The median follow-up duration was 41 months. The survival probabilities at the end of the observation period were 74.72% and 78.93% in the HIV-positive and HIV-negative groups, respectively. Overall, the HIV-positive group demonstrated a significantly worse survival outcome (HR = 1.422; 95% CI: 1.101-1.837; *P *= .033) than the HIV-negative group (Figure 2).

### Impact of comorbidities on mortality

**Figure 3.**
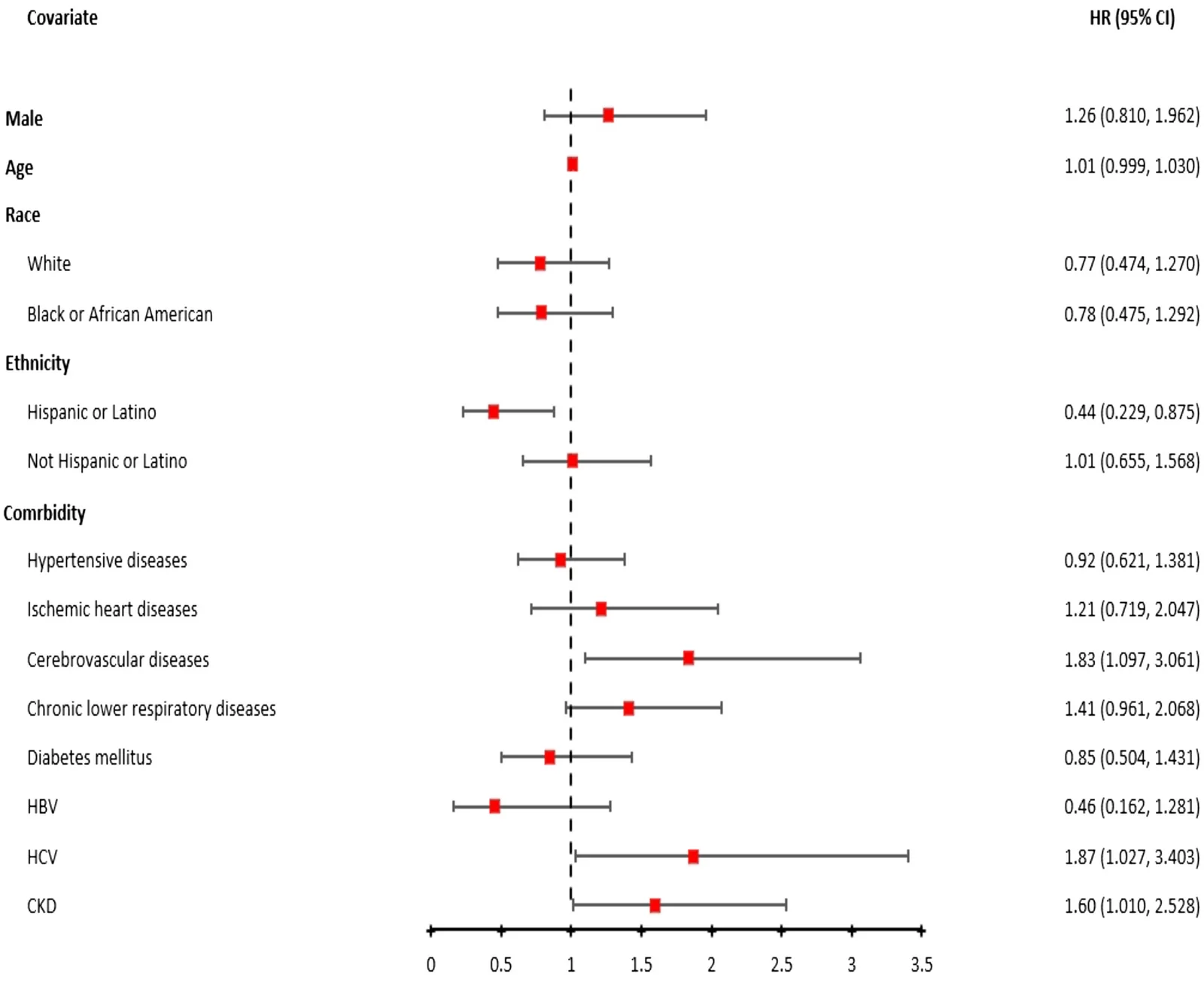
Risk factors for mortality in HIV-infected patients with Hodgkin lymphoma. Multivariable Cox proportional hazards analysis identified cerebrovascular disease (HR 1.83; 95% CI 1.10-3.06; *P* = .021), hepatitis C virus infection (HR 1.87; 95% CI 1.03-3.40; *P* = .041), and chronic kidney disease (HR 1.60; 95% CI 1.01-2.53; *P* = .045) as independent predictors of higher mortality. Conversely, Hispanic or Latino ethnicity was associated with lower mortality (HR 0.44; 95% CI 0.23-0.88; *P* = .019). CKD = chronic kidney disease; HBV = hepatitis B virus; HCV = hepatitis C virus; HR = hazard ratio.

To further explore the factors influencing prognosis, we performed a multivariable Cox proportional hazards analysis. The model revealed several comorbidities as independent predictors of mortality in HIV-infected patients with Hodgkin lymphoma (Figure 3). Cerebrovascular disease (HR = 1.83; 95% CI: 1.097-3.061; *P *= .021), hepatitis C virus infection (HR = 1.87; 95% CI: 1.027-3.403; *P *= .041), and chronic kidney disease (HR = 1.60; 95% CI: 1.010-2.528; *P *= .045) were each significantly associated with higher mortality. Conversely, Hispanic or Latino ethnicity was associated with a lower mortality risk (HR = 0.44; 95% CI: 0.229-0.875; *P *= .019). These findings suggest that comorbid conditions contribute substantially to survival disparities in patients with HIV-associated Hodgkin lymphoma.

### Impact of HIV status on survival among patients treated with ABVD chemotherapy

**Figure 4.**
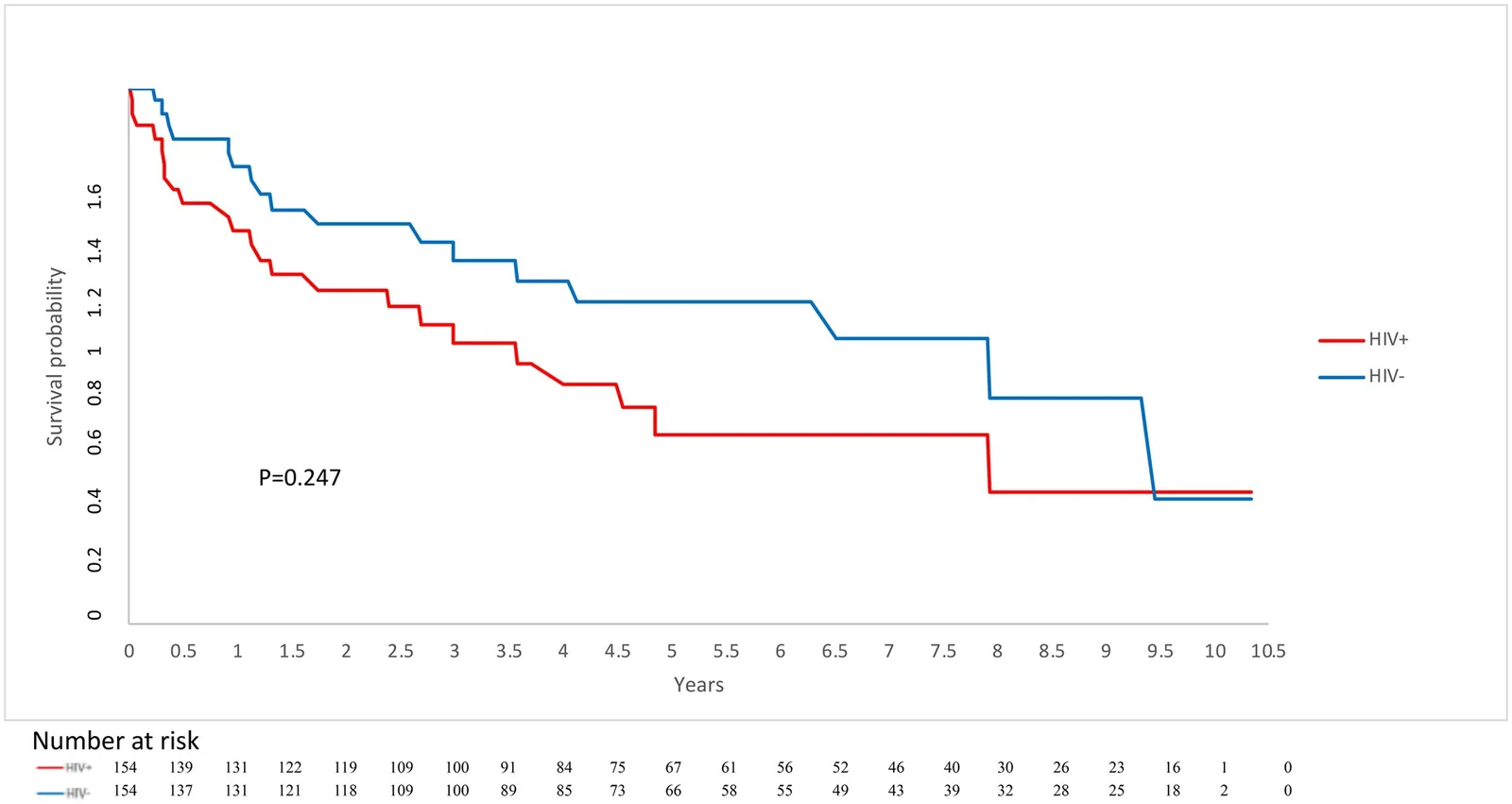
Kaplan-Meier analysis of overall survival of HIV-infected and non-HIV-infected patients with Hodgkin lymphoma treated with ABVD after propensity score matching. A total of 154 HIV-infected and 154 non-HIV-infected patients with Hodgkin lymphoma who received ABVD chemotherapy were included after 1:1 propensity score matching. During follow-up, 23 and 17 deaths occurred in the HIV-positive and HIV-negative groups, respectively. ABVD = doxorubicin, bleomycin, vinblastine, and dacarbazine.

To evaluate the impact of HIV status on treatment outcomes, we compared the survival outcomes among patients receiving ABVD chemotherapy. We performed a Kaplan-Meier survival analysis of patients with Hodgkin lymphoma in the HIV-positive and HIV-negative groups who received ABVD chemotherapy after propensity score matching. In total, 154 patients were included in each group. During the follow-up period, 23 and 17 deaths occurred in the HIV-positive and HIV-negative groups, respectively. The survival probabilities at the end of the observation period were 80.50% and 80.21% in the HIV-positive and HIV-negative groups, respectively. Overall, survival did not differ significantly between the 2 groups receiving standard treatment (HR = 1.37; 95% CI: 0.730-2.559; *P *= .247) (Figure 4). These findings suggest that HIV infection does not adversely affect the survival of patients receiving standard ABVD therapy.

### Distribution of non-ABVD first-line regimens

Despite the incomplete documentation of treatment details across the entire cohort, we managed to analyze the alternative treatment patterns between the 2 populations. Among patients not receiving standard ABVD, the selection of alternative therapies was comparable. In the HIV-positive group, 54 patients received AVD (doxorubicin, vinblastine, and dacarbazine, representing bleomycin omission) and 36 patients received the brentuximab vedotin-based regimen (BV-AVD), while none (*n* = 0) were treated with highly toxic BEACOPP-like (bleomycin, etoposide, doxorubicin, cyclophosphamide, vincristine, procarbazine, and prednisone) regimens. In comparison, within the HIV-negative group, 1565 patients received AVD, 1086 received BV-AVD, and 61 were treated with BEACOPP or BEACOPP-like therapies.

## Discussion

We performed a global retrospective cohort study using the TriNetX platform to evaluate survival outcomes in patients with Hodgkin lymphoma, stratified by HIV status and adjusted using propensity score matching. The OS was significantly better in the HIV-negative group (HR = 1.42), and cerebrovascular disease, HCV infection, and chronic kidney disease emerged as independent risk factors for increased mortality.

In our sub-cohort analysis (Figure 4), the OS curves for ABVD-treated patients remained statistically equivalent despite a numerical separation. Importantly, the HR in this subgroup (HR = 1.37) was remarkably consistent with that of the overall cohort (HR = 1.42), indicating that the underlying risk effect of HIV on survival persists. Therefore, this lack of statistical significance should be interpreted as a reflection of limited statistical power rather than true clinical equivalence. The strict selection cascade and propensity score matching caused substantial sample size attrition, reducing the number of survival events and widening CIs, leaving the subgroup analysis underpowered to detect this stable effect size. Future larger-scale collaborations are required to overcome this power limitation.

Since 2015, HIV care guidelines have recommended ART for all individuals with HIV infection and a detectable viral load, regardless of CD4 count, with the goals of improving longevity, enhancing quality of life, and reducing the risk of transmission.^2^ Accordingly, we initiated our study in 2015 to minimize bias related to patients who had not yet received ART. The widespread adoption of ART has substantially improved survival among patients with Hodgkin lymphoma, largely through the reduction of opportunistic infections and the increased feasibility of administering more intensive chemotherapy regimens.^15-17^ Meanwhile, chronic viral infection may compromise T-cell function within the tumor microenvironment, which could partly explain the inferior survival observed in patients with HIV infection with Hodgkin lymphoma.^18^ In our analysis, the OS in the HIV-positive group was significantly worse, with a 1.42-fold higher risk of death compared to that in the HIV-negative group. This finding aligns with large-scale studies, including analyses from the US National Cancer Database, which reported a 1.47-fold increased risk of mortality in HIV-infected individuals compared with the general population.^19^ Similarly, data from the ICONA cohort and affiliated European centers demonstrated a 2.37-fold higher risk of death in HIV-infected patients with Hodgkin lymphoma compared with their non-HIV-infected counterparts.^20^

Among patients receiving ABVD chemotherapy, OS was comparable between the HIV-positive and HIV-negative groups. This supports the notion that, with effective ART and standard chemotherapy, HIV-infected patients with Hodgkin lymphoma can achieve outcomes similar to those of their non-HIV-infected counterparts. Additionally, although most of our HIV-positive group (72.5%) received timely ART, a subgroup analysis stratifying survival outcome by ART status was precluded by the limited sample size and the low number of survival events within the non-ART subgroup (*n* = 255). Consistent results have been reported for several cohorts in the ART era. A UK-based cohort study of 224 patients (93 HIV-positive) showed 5-year OS rates of 81% vs 88% in the HIV-positive and HIV-negative groups, respectively, with no significant difference.^11^ The German HIV-Related Lymphoma Study Group similarly reported a long-term OS of 76.1%-95.7% in the HIV-positive group primarily treated with ABVD, which was comparable to that in the HIV-negative group.^21^ A Spanish retrospective analysis found a 5-year OS of 81% and 88% in the HIV-positive and HIV-negative groups, respectively, with no significant difference in complete response rates.^22^ In the French ANRS-CO16 Lymphovir cohort, HIV-infected patients treated with ART and predominantly ABVD also achieved survival outcomes comparable to those of non-HIV-infected patients.^10^ Collectively, these findings reinforce that, in the ART era, HIV infection is not a barrier to favorable outcomes when standard chemotherapy is appropriately delivered.

The inferior outcomes observed in the overall HIV-positive cohort compared with those receiving ABVD may reflect disparities in access to cancer care. Patients with HIV infection are less likely to be offered or to complete standard chemotherapy because of comorbidities, concerns about toxicity, delayed diagnosis, or stigma-related barriers within the healthcare system. The HIV/AIDS Cancer Match Study reported that HIV-infected individuals were 1.39 times more likely to forgo treatment for Hodgkin lymphoma, underscoring disparities in care.^13^ Similarly, a national study from the Moffitt Cancer Center found that 16.5% of HIV-infected patients did not receive recommended first-line curative therapy. This treatment gap is strongly associated with lower income and education levels.^23^ These findings highlight persistent inequities in cancer care for people living with HIV, which likely contribute to poorer outcomes despite the availability of effective therapies.

Our data on alternative first-line therapies provide important insights into the real-world management of HIV-associated Hodgkin lymphoma. The omission of bleomycin (resulting in the AVD regimen, utilized in 54 patients in our HIV cohort) is a well-established strategy to prevent cumulative pulmonary toxicity, which carries a higher mortality risk in PLWH due to a predisposition to opportunistic lung infections. Moreover, the adoption of BV-AVD in 36 HIV-positive patients highlights the increasing integration of targeted antibody-drug conjugates in modern protocols, offering excellent efficacy while reducing bleomycin-related lung injury. However, the complete absence of BEACOPP utilization (*n* = 0) in our HIV cohort shows that clinicians avoid highly myelosuppressive regimens in this immunologically vulnerable population to prevent severe neutropenic sepsis and treatment-related mortality.

Our study identified cerebrovascular disease, HCV infection, and CKD as independent predictors of increased mortality among HIV-positive patients with Hodgkin lymphoma. HIV/HCV coinfection has been linked to a 65% higher risk of all-cause mortality in large ART-era cohorts.^24^ Similarly, CKD has been shown to increase the all-cause mortality risk by 1.58-fold in patients with lymphoma.^25^ Although direct evidence of cerebrovascular disease in HIV-positive Hodgkin lymphoma is lacking, it is plausible that this comorbidity exacerbates systemic inflammation, reduces treatment tolerance, and contributes to poorer outcomes. Collectively, these comorbidities may underlie the survival disparities in patients with HIV with Hodgkin lymphoma, underscoring the need for the comprehensive management of coexisting conditions.

Other frontline regimens, including BEACOPP or BV-AVD, are also considered treatment options for Hodgkin lymphoma.^26^^,^^27^ However, BEACOPP is more frequently used in European countries, and the number of patients receiving BV-AVD in our cohort was small, limiting our ability to conduct a meaningful comparison with the ABVD regimen in this study.

This study had several limitations. First, key clinical parameters such as CD4 cell counts, HIV viral load, and disease stage were not analyzed because fewer than 10% of these data were available in the TriNetX database, limiting the assessment of immune status and viral suppression of outcomes. However, prior studies have suggested that HIV status alone does not significantly affect the prognosis of patients with Hodgkin lymphoma treated with ABVD, which may mitigate this limitation.^11^ For the clinical stage, which is a key predictor of outcomes in Hodgkin lymphoma, was largely unavailable in our dataset. Our data revealed that only 2.3% (1184/50 903) of the total population possessed staging records (Stage I: *n* = 169, Stage II: *n* = 474, Stage III: *n* = 282, Stage IV: *n* = 284). Due to this high rate of missing data, forcing stage into the propensity score matching algorithm would have severely limited the sample size, reducing statistical power and introducing substantial selection bias. Second, the number of patients with documented ABVD chemotherapy was relatively small, largely due to incomplete treatment records in TriNetX. Both the HIV-positive and HIV-negative groups experienced substantial reductions in sample size when the analyses were restricted to patients with verifiable ABVD administration. Importantly, the excluded patients were not necessarily untreated but may have lacked complete documentation. To ensure analytical accuracy, only patients who received standard chemotherapy regimens were included. Third, given the limited number of ABVD-treated patients, we were unable to conduct further subgroup analyses based on chemotherapy dose or number of cycles. Although such analyses could provide insights into treatment intensity and outcomes, the current sample size does not allow for reliable stratification. In real-world, HIV-infected patients often require dose modifications or experience treatment delays due to chemotherapy-induced toxicities or drug-drug interactions with ART. Consequently, potential confounding relative to dose intensity or regimen completion rates cannot be completely excluded. Forth, due to the inherent nature of the TriNetX registry and electronic health records, the oncological endpoints such as progression-free survival and lymphoma-specific survival were not available. Consequently, misclassification of progression events or death from non-lymphoma-related causes cannot be entirely ruled out. However, OS (all-cause mortality) used in this study serve as an indicator of clinical outcomes due to high grade disease nature. Our findings should be interpreted with caution, and future prospective trials capturing disease-specific outcomes are required to confirm these observations. Fifth, although TriNetX is a global network, approximately 91% of our study cohort was sourced from United States healthcare organizations. Consequently, the findings of this study may lack global generalizability, and caution should be exercised when extrapolating these results to non-US healthcare settings with different socioeconomic or epidemiological profiles.

In conclusion, our study demonstrates that although OS is lower in HIV-infected patients with Hodgkin lymphoma, those treated with standard ABVD chemotherapy achieve outcomes comparable to those of non-HIV-infected patients. These findings underscore the need to ensure equitable access to guideline-based cancer therapy for PLWH. Strengthening cancer care delivery and allocating adequate healthcare resources to this population should be prioritized.

## Data Availability

The data underlying this article will be made available by the corresponding author to qualified researchers upon reasonable request. For original data, please contact [ccwei@vghtc.gov.tw].
